# Editorial: Digital collaborative learning in general, higher, and business education

**DOI:** 10.3389/fpsyg.2025.1572277

**Published:** 2025-03-27

**Authors:** Henrik Bellhäuser, Christin Siegfried, René Röpke

**Affiliations:** ^1^Psychology in Educational Sciences, Johannes Gutenberg University Mainz, Mainz, Rhineland-Palatinate, Germany; ^2^Business Education for Vocational Teacher Education, Potsdam University, Potsdam, Brandenburg, Germany; ^3^Learning Technologies and eDidactics Research Lab, Vienna University of Technology, Vienna, Austria

**Keywords:** collaborative learning, cooperative learning, collaborative working, cooperative working, digital learning, digital working, digital collaboration, digital cooperation

Virtual collaboration and digital learning in higher education have become pivotal in recent years, fueled by rapid advancements in technology and an increasing emphasis on interdisciplinary approaches to problem-solving. While cooperative working or learning in general contributes to with the expectation that people will achieve more together than alone (Järvelä et al., 2015; Johnson et al., 2000; Rosé et al., 2008), if there is an appropriate composition of teams and the participation of each team member through communication (written, verbal, or non-verbal), in the form of diverse and elaborate sharing of ideas, experiences and knowledge (Bellhäuser et al., 2018; Müller et al., 2024a,b; Siegfried, 2021; Tsovaltzi et al., 2019). The advent of the World Wide Web and other digital technologies has significantly transformed cooperation. The Internet's constant connectivity enables individuals to engage with peers—including family, friends, and colleagues—at any time and from anywhere through various communication media, apps, and web-based tools (Lane et al., 2024). Moreover, asynchronous collaboration, supported by digital tools for recording and revising speech and writing, encourages thoughtful reflection before contributions are shared with the team (Chi, 2009). These technologies also facilitate the externalization of thought processes by allowing individuals to structure and visualize their ideas, making them more accessible and comprehensible to team members (Jessop, 2008). Additionally, digital collaboration helps reduce social isolation, a prevalent challenge in virtual learning environments, while enhancing satisfaction by fostering meaningful interaction and engagement (Efimov et al., 2022).

At the same time, the potentials of digital cooperative working and learning mentioned above are not (or cannot) always be realized. Reasons for this lie not only in often inadequate technical equipment (Jeong and Hmelo-Silver, 2016), but also in the competencies required by this new form of cooperation, which are not or insufficiently trained on the part of the users (Beek et al., 2020; Theobald et al., 2023). So far, however, there have only been isolated findings with regard to the potential and the challenges of digital collaborative learning and working.

Accordingly, this Research Topic aims to bring together research from different disciplines. Thus, the collection of papers in this Research Topic explores a variety of themes surrounding virtual collaboration and digital learning in higher education, with a focus on the challenges, strategies, and outcomes associated with the use of digital platforms for collaborative learning and teamwork. By looking at the dynamics of interdisciplinary collaboration, the contributions highlight both the potential and limitations of digital environments for fostering meaningful learning experiences.

For instance, one paper emphasizes the difficulties faced by interdisciplinary academics in adapting to virtual collaboration, noting issues such as technical limitations, management challenges, and cultural differences that influence virtual teamwork dynamics.

Some studies explore how specific preparation tasks or communication styles impact learning outcomes. For example, one paper examines how different levels of generative preparation tasks, such as note-taking and explanation activities, affect students' deep comprehension during digital collaborative learning, finding that prior knowledge and task structure play significant roles in learning outcomes. Similarly, another study investigates the relationship between students' achievement goals and collaborative activities, revealing that learning-oriented goals enhance students' ability to sequentially organize collaboration efforts, ultimately improving knowledge acquisition.

The influence of personality traits and social dynamics in collaborative environments is also addressed. One paper uses the TREO framework to show how individual personality traits and team roles affect communication patterns in collaborative problem-solving tasks, while another study focuses on non-verbal behaviors, such as nodding and leaning forward, to understand how these actions foster engagement in virtual learning.

Some studies focus on specific groups or contexts, like non-traditional students facing social identity threats in computer-supported collaborative learning (CSCL) environments. These students' sense of belonging and motivation are challenged by stereotypes, impacting their engagement and collaboration effectiveness. Another paper examines doctoral students' experiences in virtual communities of practice, emphasizing the role of distributed leadership, shared goals, and collaborative support in enhancing remote learning experiences.

Furthermore, the papers address practical implications, such as the need for digital competences and technological-pedagogical knowledge. Tools and methods, such as coding manuals and content analysis, are developed to quantify transactive communication and problem-solving activities, providing insights into students' cognitive and metacognitive engagement in tasks like glossary creation and concept mapping.

Overall, the studies presented in this Research Topic contribute to understanding how digital collaborative environments affect learning outcomes, highlighting the importance of structured tasks, social dynamics, and technological support in maximizing the effectiveness of virtual collaboration in education.
